# Characteristics of adolescents who expressed indifference or no interest towards body art

**DOI:** 10.1186/1471-2458-10-605

**Published:** 2010-10-13

**Authors:** Luca Cegolon, Carla C Xodo, Giuseppe Mastrangelo

**Affiliations:** 1 Padua University, Department of Environmental Medicine and Public Health, Padua, Italy; 2 Imperial College London, School of Public Health, St Mary's Campus, London, UK; 3 Padua University, Department of Educational Sciences, Padua, Italy

## Abstract

**Background:**

This study examines the underlying characteristics of adolescents attending Italian secondary school who expressed indifference towards or no interest at all in body art.

**Methods:**

A convenience sample of 4,277 secondary school students from the North Eastern Italy were surveyed with a self-reported questionnaire collecting extensive socio-demographic information. Multivariable logistic regression analysis was employed to investigate the characteristics of those who were not interested or indifferent towards piercing and tattoo, reporting adjusted Odds Ratios (aOR) with a 95% confidence interval (CI).

**Results:**

Prevalence of tattoo was 6%, whereas body piercing was 20%; 66% (= 558/840) of those with a piercing were underage (<18 years of age), the equivalent for tattoo being 62% (= 159/258). 166 individuals reported having both piercing and tattoo and 152 of these (92% = 152/166) were <18 years of age. The factors found to be predominately higher in those indifferent or who did not indicate interest in body art were: higher school year, increasing father's education and a greater perception of the associated health risks.

**Discussion:**

Proactive health education campaigns by school educators and family physicians should focus on adolescents of less educated fathers and pupils less aware of the health risks associated with body art. In this respect junior secondary school students can be regarded as the ideal target of such campaigns.

## Background

Body art (piercing and tattoo) is currently reported as being all the rage worldwide [1-4], with a prevalence estimated to range from between 10% [1] and 51% [5] for piercing and 4.5% [6] to 24% [7] for tattooing.

Despite the prevalence of body modification, especially among adolescents [1,5,8] and the associated health risks [1,9-12], these practices are currently not sufficiently regulated [13-17]. It has been reported, moreover, that a substantial percentage of girls and boys were not aware of the associated risks and cautions to be observed when deciding to undertake body modification [18]. A recent study conducted in Italy [8] investigating the average adolescent in a standard secondary school showed a high percentage of adolescents actually desiring body modification, and this percentage was far higher than the percentage of those already having undergone body modification. There is therefore a need to provide education to better assist their decision making regarding any body art purchases.

Since pupils not interested or indifferent towards these practices were a small minority in the above survey [8,18], in the present study they have been compared with those having a positive attitude towards tattoos or piercing. The aim of the study is to better understand the characteristics differentiating these two distinct groups in order to frame future health education programs particularly in light of the fact that there has been very little prior research into this area.

## Methods

### Sampling Strategy

Education is currently obligatory until the age of 16 in Italy and pupils begin secondary school at the age of 13-14, after leaving junior secondary school. Grouped into separate sections, students attend secondary school for 5 years, thus normally completing school at the age of 18-19, unless repetition of one or more school years is required for unsatisfactory performance.

This study was conducted in the Veneto Region (Northeast of Italy), where 6 schools (corresponding to each of the six types of Italian public secondary schools) were selected in each of the 7 Provinces of the Region. The schools were chosen on the basis of individual negotiations with the respective head-teachers, and out of the total number of 42 (= 6 × 7) schools, 41 eventually agreed to take part in the study. Among two sections of pupils randomly selected in each school, adolescents attending the 1^st^, 3^rd^, and 5^th ^school years were enrolled, for a final target population of 4,524 students aged 13-21 years.

### The Survey

The field survey was conducted in 2007, during regular morning classes, under the permission of the teacher and the school head-teacher. Before distributing self-administered questionnaires, a researcher explained purpose/methods of the study and the time necessary to complete the interview (approximately 10 minutes). The researcher also confirmed to the pupils and the teacher that participation in the survey was voluntary and confidential.

A structured 22-item questionnaire accessible elsewhere [18] included questions on place of residence ("city" any Province capital; "town" >15,000 inhabitants; "small town" <15,000 inhabitants); Province of residence; single parent household; number of siblings; sex and age of each sibling; father's/mother's age (at the time of the interview); educational level of father/mother (low = junior secondary school, corresponding in Italy to going to school until 13 years of age; medium = secondary school; high = university or postgraduate degree); satisfaction with physical appearance (yes, fairly, no); attitude towards tattoo and piercing separately (indifferent or not interested, interested or keen to try, already experienced). Unlike boys, body piercing on a girl was defined as any piercing of the body, excluding the earlobe, as it is largely common.

A preliminary pilot study was conducted on a convenience sample of 100 secondary school students of the same Region to test and eventually adjust the questionnaire.

The study design and protocol were approved by the schools' head-teachers and the Postgraduate Training Institution for Secondary School Teachers of the Veneto Region (SSIS Veneto, an educational body part of the University of Padua), which also provided the ethical approval. Parental consent for participants younger than 18 years was considered unnecessary by the SSIS Veneto, as the questions of the survey were not intrusive and full confidentiality was guaranteed given the questionnaire was anonymous and self-administered.

The response rate was 100%, but due to some of the questionnaires having to be discarded (illegible, non-sensical), the final number of observations was reduced to 4,277, 95% (= 4,277/4,524) of the initial number.

### Statistical Analysis

The outcome variable was 1 if the respondent was not interested or indifferent to any of these practices, and 0 otherwise. The independent variables included several personal and family background characteristics as well as the following five items derived from the questionnaire:

1. "*Infectious diseases knowledge*" which was 1 (and 0 otherwise) if the student answered "yes" to the question: "Do you think there are diseases associated with the practice of tattoo or piercing?" and ticked at least five of the 10 pre-classified responses to the question "Which of the following diseases can be transmitted by piercing or tattoo?";

2. "*Knowledge of mandatory hygienic rules related to body art*" which was 1 (and 0 otherwise) if the student gave maximum priority to "adoption of single-use needles; systematic sterilization of equipment; use of latex gloves" in answering the question "Please identify the indicators of professionalism that a body art operator should have";

3. "*Propensity to refer to a professionally certified body art practitioner*", which was 1 (and 0 otherwise) 1 if the respondent agreed. In Italy individuals interested in performing body art legally are required to attend a professional course organised by the Regional Public Health Department. The subjects taught in these courses span from physiopathology of the skin to hygiene and preventative medicine. The single Primary Care Trusts (PCTs) are responsible for the surveillance of these body art operators and parlors [19];

4. *"Propensity to seek medical advice in the event of complications*", which was 1 (and 0 otherwise) if the respondent agreed;

5. "*Awareness of the implications of tattoo removal*", which was 1 (and 0 otherwise) if the student answered: "*I am aware it would be difficult (to remove)*" or "*I do not think I will try to remove it*" to the question "*Do you consider the difficulties involved in the removal of tattoos*?"

Multivariable logistic regression analysis, reporting adjusted Odds Ratios (aOR) with 95% confidence interval (CI), was built up selecting terms by backwards stepwise selection. Since this is a secondary analysis using data from a project already partially reported [8,18], the p-value was downsized (p < 0.01 from the previous p < 0.05).

Missing data were excluded and complete case analysis was performed with Stata 11 (Stata Corporation, College Station, Texas, USA).

## Results

Table 1 shows data already partially reported [8,18]. Strata were of roughly similar size in respect of age, school year, province of residence, age of mother and father and satisfaction with physical appearance. Most students were females (65%), lived in small towns, in families with both parents, with more than two children and of a low or medium level of education level of their respective parents. Prevalence of tattoo was 6% (= 258/4,086), whereas body piercing was 20% (= 840/4,177); 25% of the un-pierced considered piercing, 47% of the non-tattooed considered tattoo. Sixty-six percent (= 556/840) of those with a piercing were <18 years of age, the equivalent for tattoo being 62% (= 159/258). One hundred sixty-six individuals reported having both piercing and tattoo and 152 of these (92% = 152/166) were <18 years of age. Seventy-four percent (= 2,997/4,069) of the respondents would refer to a health care professional in case of complications associated with body modifications, 72% (= 2,803/3,879) had a sufficient knowledge of these hygienic norms, 64% (= 2,714/4,277) considered it important to refer to a certified body art parlor, 54% (= 1,820/3,347) had a reasonable knowledge of the infectious diseases related to body art, while only 40% of the respondents seemed aware of the problems associated with tattoo removal.

**Table 1:** Frequency distribution of 4,277 secondary school pupils by explanatory and outcome variables

			OUTCOME	
TERMS		No. (%)	YES (No; %)	NO (No.; %)
**Gender**	Female	2,789 (65.2)	821 (54.6)	1,860 (71.7)
	Male	1,488(34.8)	683 (45.4)	734 (28.3)

	<15	1,494 (34.9)	565 (38.6)	854 (32.4)
**Age (years)**	16-17	1,501 (35.1)	473 (31.2)	969 (37.7)
	18+	1,282 (30.0)	466 (30.2)	770 (29.9)

**School year**	1^st^	1,566 (36.6)	604 (40.2)	886 (34.2)
	3^rd^	1,478 (34.6)	466 (32.0)	961 (37.1)
	5^th^	1,181 (28.8)	434 (28.9)	747 (28.8)

	City centre	850 (20.7)	297 (20.6)	520 (20.9)
**Residence **(missing: 179)	City outskirt	979 (23.9)	347 (24.1)	589 (23.7)
	Town	412 (10.1)	164 (11.4)	236 (9.5)
	Small town	1,857 (45.3)	631 (43.9)	1,144 (45.9)

	Belluno	509 (12.0)	203 (13.6)	287 (11.2)
	Verona	674 (15.9)	222 (14.8)	427 (16.6)
	Vicenza	402 (9.5)	116 (7.8)	272 (10.6)
**Province of residence **(missing: 32)	Padua	739 (17.4)	285 (19.1)	433 (16.8)
	Venice	554 (13.1)	215 (14.4)	320 (12.5)
	Treviso	621 (14.6)	222 (14.8)	369 (14.3)
	Rovigo	746 (17.6)	233 (15.6)	463 (18.0)

**Satisfaction with physical appearance **(missing 69)	Yes	1,511 (35.9)	615 (41.4)	839 (32.7)
	Fairly	2,258 (53.7)	759 (51.2)	1,418 (55.2)
	No	439 (10.4)	110 (7.4)	310 (12.1)

**Single parent household**	No	3,806 (89.0)	1,371 (91.2)	2,272 (87.6)
	Yes	471 (11.0)	133 (8.8)	322 (12.4)

**No. of siblings **(95 missing)	0	779 (18.6)	279 (18.9)	463 (18.2)
	1	2,349 (56.2)	823 (55.9)	1,439 (56.6)
	2+	1,054 (25.2)	371 (25.2)	639 (25.2)

**Senior sibling of same sex**	Yes	3,963 (92.7)	1,356 (90.2)	2,441 (94.1)
	No	314 (7.3)	148 (9.8)	153 (5.9)

**Father's age (years) **(missing: 409)	<47	1,542 (39.9)	509 (37.1)	973 (41.6)
	48-51	1,107 (28.6)	383 (28.0)	677 (28.9)
	52+	1,219 (31.5)	478 (34.9)	692 (29.5)

**Mother's age (years) **(missing: 337)	<44	1,411 (37.3)	485 (34.9)	926 (38.7)
	45-48	1,149 (30.3)	403 (29.0)	746 (31.2)
	49+	1,223 (32.4)	502 (36.1)	721 (30.1)

**Mother's education **(missing: 144)	Low	1,456 (35.2)	474 (32.4)	929 (37.1)
	Medium	2,007 (48.6)	712 (48.7)	1,213 (48.4)
	High	670 (16.2)	276 (18.9)	363 (14..5)

**Father's education **(missing: 216)	Low	1,353 (33.3)	416 (28.8)	886 (36.1)
	Medium	1,917 (47.2)	699 (48.4)	1,149 (46.9)
	High	791 (19.5)	329 (22.8)	417 (17.0)

**Attitude towards piercing **(missing: 100)	Indifferent/Not interested	2,276 (54.5)		
	Interested/Keen to try	1,061(25.4)		
	Done	840 (20.1)		

**Attitude towards Tattoo **(missing: 191)	Indifferent/Not interested	1,900 (46.5)		
	Interested/Keen to try	1,928 (47.2)		
	Done	258 (6.3)		

**Infections related knowledge **(missing: 1,070)	Lower	1,210 (37.7)	407 (35.6)	769 (39.3)
	Higher	1,997 (62.3)	737 (64.4)	1,189 (60.7)

**Hygienic rules knowledge **(missing: 398)	No	1,076 (27.7)	369 (29.2)	659 (26.6)
	yes	2,803 (72.3)	895 (70.8)	1,815 (73.4)

**Propensity for professionally certified parlors**	No	1,563 (36.5)	563 (37.4)	917 (35.4)
	Yes	2,714 (63.5)	941 (62.6)	1,677 (65.6)

**Propensity for health care professionals **(missing: 841)	No	924 (26.9)	182 (22.0)	738 (28.5)
	Yes	2,512 (73.1)	646 (78.0)	1,856 (71.6)

**Awareness of difficulties in tattoo removal **(Missing: 191)	No	2,462 (60.2)	1,283 (85.3)	1,172 (45.5)
	Yes	1,624 (39.8)	221 (14.7)	1,403 (55.5)

(YES = being indifferent/not interested to both piercing and tattooing; NO = being Interested/keen to try or having already done at least one of these practices); Number (No.) and percentages (%).

Table 2 shows the results of the final multivariable logistic regression model, fitted on 2,071 complete observations. Those more likely to be indifferent or not interested in either piercing or tattoo were pupils attending the fifth year of school, with higher knowledge of the infections related to body art, and higher propensity to refer to a certified parlor to receive body art or to a health care provider in case of related medical complications. By contrast, adolescents indifferent/not interested in body art were less aware of the implications of tattoo removal. Lastly, the likelihood of being not interested/indifferent towards body art shows a significant increasing trend with the higher the father's education level.

**Table 2:** Multivariable logistic regression model fitted on 2,071 complete observations.

TERMS		aOR (99% CI)
	1^st^	*Reference*
**School Year**	3 ^rd^	NS*
	5 ^rd^	0.72 (0.58 to 0.91)

	Low	*Reference*
**Father's educational level**	Medium	1.41 (1.11 to 1.80)
	High	1.74 (1.29 to 2.35)

	Lower	*Reference*
**Infections related knowledge**	Higher	1.29 (1.04 to 1.62)

**Propensity for certified body art parlors**	No	*Reference*
	Yes	`1.46 (1.14 to 1.85)

	No	*Reference*
**Propensity for health care professionals**	Yes	1.57 (1.20 to 2.06)

**Awareness of the implications of tattoo removal**	No	*Reference*
	Yes	0.34 (0.27 to 0.42)

Adjusted Odds Ratio (aOR), and 99% Confidence Interval (99% CI).

* Non Significant

## Discussion

The role of the father has seldom been mentioned in the available literature. In two national surveys of nearly 3,000 adolescents, 47% of the 318 youths with tattoos had not told their parents about their art and 86% did not obtain parental consent [20]. 137 career-oriented women who had had tattoos for at least six months prior to being interviewed perceived strong support for the tattoo from their friends, mild support from their mothers and siblings, whereas respondents cited a lack of, or negative response from their fathers [21]. A study of Norwegian teenagers found that support from parents and closeness to them was lower in those who had had body modifications than in those without [22]. Men (or women) with body modifications were more likely to make their parents angry compared with men (or women) without modifications. Also men with modifications were less close to their fathers than men without modifications [23].

In the present study children of a more educated father were less likely to be interested in body art. Likewise, according to Roberts [6], the proportion of adolescents with tattoos decreased with the increasing level of parental education reported by either parent. Given their higher educational level, the fathers' role might be cultural and educational, not necessarily repressive. Though, the nature of the background and upbringing that more highly educated fathers transmit to their children is unknown. Further research appears to be warranted in order to fully understand this influence. Meanwhile, fathers should be proactively encouraged to provide information, and possibly dissuade their children from practices often undertaken to comply with a social fashion.

Previous findings from the same survey reported that adolescents of less educated fathers had a positive attitude towards body piercing [8]. It may be possible that less educated fathers support their children in undergoing body modifications, possibly even sharing their positive experiences of it. It could also be the case that adolescents desiring body modification might be stimulated by their respective fathers.

The present results - suggesting that the mother appears not to be influential in the decision of the child to undertake body modification - align with other findings from the same survey [8,18] as well as with those from other studies [20,23].

Earlier results from the same research survey [Cegolon L et al. "*Tattoo removal in the average adolescent*", submitted] showed how adolescents with a positive attitude towards body art (interested in or having already a piercing or tattoo) were more likely to be aware of the implications of removing a tattoo, as compared with those indifferent or not interested. By contrast, in the present study we found that the lack of interest in body art was significantly associated with a higher perception of the associated health risks. Consequently we feel that an eventual health education program should also incorporate information about the health risks associated with body art in an attempt to educate more youngsters of the risks and thereby reduce its incidence.

It has been reported that adolescents undertake body modifications despite being mostly composed of minors [8,23-25]. Present findings suggest that the interest in body art seems to increase with increasing school years. Therefore, in order to counterbalance peer influence/support amongst youngsters, which is a known factor causing experimentation in this area [26], a proactive health education campaign should be framed. Currently there is a lack of education on these risks in Italy and the present study is evidence of the need to provide consistent educational programs on this subject. In this respect junior secondary school students can be regarded as the ideal target of such campaigns. Since both education and health information is needed, a best strategy could probably entail a coordination of school educators/counselors, parents (especially fathers) of younger adolescents, and health care providers (school nurses and particularly primary care practitioners).

Strengths and weaknesses of this research survey have been reported elsewhere [18], the main limitation being the convenience rather than random sampling of schools and/or students. However, in view of the considerable numbers involved, we believe our findings could be reasonably applied to any westernized society.

## Conclusions

Relevant health education programs should be guided and oriented by various figures such as school educators/counselors, school nurses, primary care practitioners, all types of physicians and others who are in contact with teenagers. This multi-agency team of key professionals should work in coordination with each other and the children' families.

## Competing interests

The authors declare that they have no competing interests.

## Contributors

LC participated in the design of the study, the statistical analysis and the drafting of the paper; GM contributed to the design of the study, the statistical analysis and the drafting of the paper; CX participated in the design of the study and is the guarantor. All authors read and approved the final manuscript.

## Pre-publication history

The pre-publication history for this paper can be accessed here:

http://www.biomedcentral.com/1471-2458/10/605/prepub
